# Prevention and Control Strategies to Counter ZIKA Epidemic

**DOI:** 10.3389/fmicb.2017.00305

**Published:** 2017-02-28

**Authors:** Irfan A. Rather, Sanjay Kumar, Vivek K. Bajpai, Jeongheui Lim, Yong-Ha Park

**Affiliations:** ^1^Department of Applied Microbiology and Biotechnology, School of Biotechnology, Yeungnam UniversityGyeongsan, South Korea; ^2^Department of Poultry Science, University of GeorgiaAthens, GA, USA; ^3^National Science Museum, ICT and Future PlanningYuseong-gu, South Korea

**Keywords:** ZIKA, diagnoses, prevention, treatment, awareness

## Abstract

ZIKA virus (ZIKA) has now become a global phenomenon. Since 2007, evidence of ZIKA transmission has been reported over 72 countries and territories. The transmission of ZIKA has made World Health Organization to categorize the situation under the ambit of a health emergency. This situation is serious because there appears to be a highly tangible link between infection during pregnancy and the occurrence of microcephaly and Guillain–Barré syndrome. In the context of this emergency situation, this review article intends to discuss the prevention and control strategies such as avoiding travel to infected area, being careful from mosquito bites, take precautions to reduce the risk of sexual transmission, and seek medical care for any acute illness with rash or fever. This review is an attempt to analyze the results of those campaigns, keeping in view the variables and constants that affect any such measures. Furthermore, this article will suggest proactive measures that can be employed to effectively combat the epidemic transmission of the ZIKA.

## Introduction

**FIGURE 1:**
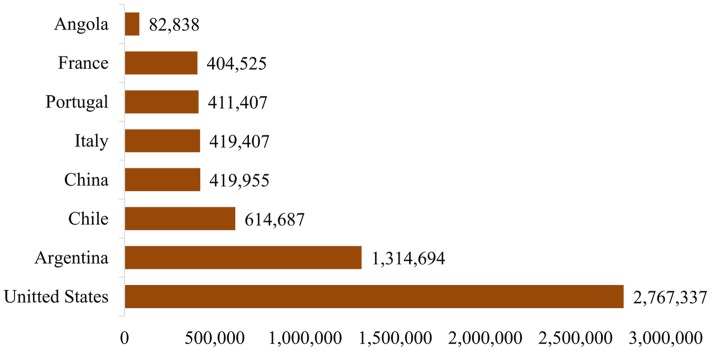
**Number of travelers to and from Brazil to other countries (Statista, 2016)**.

ZIKA is a member of family Flaviviridae and the genus *Flavivirus*. The virus is icosahedral, enveloped, non-segmented, single-stranded, 10 kb positive-sense RNA genome that closely relates to the Spondweni virus (Hayes, 2009; Faye et al., 2014; Hafiz et al., 2016). Apart from lipid bilayer and one genome RNA, ZIKA contains three distinct types of structural proteins such as envelope protein (E), M-membrane protein (M/prM) and capsid or core protein (C) and seven non-structural proteins (Lindenbach and Rice, 2003; Dia et al., 2016). ZIKA infection is caused by the bites of daytime-active *Aedes* mosquitoes such as *Aedes aegypti* and *A. albopictus* (Malone et al., 2016). The virus was first isolated from *Aedes africanus* infected rhesus monkey in 1947 and is named after the Ugandan ZIKA Forest (Dick, 1952; Weinbren and Williams, 1958). The parallel research indicated that *A. aegypti* has also the capability of transmitting ZIKA to monkeys and mice (Boorman and Porterfield, 1956). This led to the suspicion that the virus can possibly infect humans. The first major outbreak of ZIKA infection was reported from the Island of Yap in 2007 followed by Brazil in 2015. The most commonly reported symptoms of ZIKA are mild fever, skin rashes, joint pain, myalgia, and conjunctivitis (red eyes). Many people infected with ZIKA do not get sick, people who get sick usually report a number of symptoms, resembling the symptoms of dengue fever (Malone et al., 2016), with a very low mortality rate. This is the primary reason that the prevention and control strategies are rarely implemented in infected population; however, there is a need to prevent further infections. The virus attracted the spotlight from public health officials because of its highly suspected association with maternal–fetal transmission and the microcephaly in the infected fetus as well as other associated neurological abnormalities in adults with Guillain–Barré syndrome (GBS). Several prevention and control strategies have been discussed and implemented to control the epidemic. The World Health Organization (WHO) currently listed following 72 countries and territories where evidence of ZIKA transmission has been reported since 2007 (WHO, 2016). In 2015 alone, the evidence of ZIKA transmission was reported in 56 countries and territories; in 2016, it was reported in five countries, whereas in 12 countries and territories the evidence of ZIKA transmission could be in or before 2015. Further, since February 2016, 12 countries have reported evidence of person-to-person ZIKA transmission and over 12 countries reported microcephaly and other CNS malfunctions associated with ZIKA. In addition, four countries reported microcephalic babies born from mothers in countries with no endemic ZIKA transmission, but have traveled to ZIKA affected countries in the past. Therefore, to minimize the transmission, and associated effects including fetal microcephaly, the Centers for Disease Control and Prevention (CDC) circulated an advisory, particularly for the expecting women, to avoid or at least postpone their visit to the areas where transmission rate is high (Oliveira et al., 2016), since, the risk of infection could be associated with travelers from Brazil to other countries. From September 2014 to August 2015, nearly 2.8 million people travel to and from Brazil to United Stated as shown in **Figure 1** (Statista, 2016), suggesting that there is a high risk factor of ZIKA infection.

### Epidemiology

Because of the asymptomatic clinical course of ZIKA infection, it is difficult to calculate precise global prevalence. It has not been widely reported owing to its clinical resemblance with other flavivirus infections, and difficulty in differential diagnosis.

**FIGURE 2:**
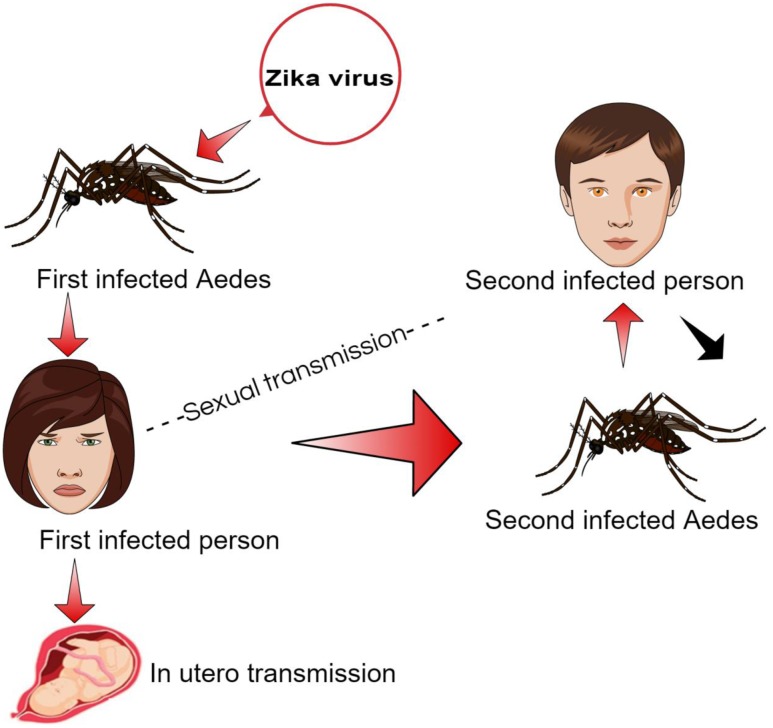
**ZIKA transmission cycle in humans**.

The ZIKA has been reported in various hosts including mosquitos, humans, and, monkeys through sporadic case reports, seroprevalence surveys and entomological surveys in 14 different countries across Africa, Asia, and Oceania (Ioos et al., 2014). The virus may also pass from primary to secondary host through blood transfusion via infected blood cells and sexual contact (**Figure 2**). The ZIKA infection symptoms usually appear in 3–12 days after the vector bite and end within 2–7 days after onset of symptoms (Duffy et al., 2009).

In South America, Brazil reported first outbreak of ZIKA around mid of 2015. Brazil Ministry of Health gave an estimated figure of 440,000–1,300,000 suspected cases of patients infected with ZIKA in December 2015 (ECDC, 2016). In the United States as of May, 2016, a total of 472 cases of ZIKA were reported that are travel-associated. In other US territories, a total of three travel-associated cases and 658 locally acquired cases were reported (CDC, 2016a). Microcephaly and congenital syndrome associated with ZIKA are also prevalent in countries, which have ZIKA outbreak as shown in **Table 1**.

**Table 1:** ZIKA microcephaly and congenital syndrome by September 2016 (WHO, 2016).

Location	Number of confirmed cases by September 2016
Brazil	1911
Colombia	40
Martinique	12
USA	23
French Polynesia	8
Panama	5

## Clinical Presentation and Diagnosis

As previously discussed ZIKA transmits to humans through infected *A. aegypti* and *A. albopictus* mosquitoes. Further, if infects pregnant mother, the virus can pass to the fetus and lead to microcephaly. In Colombia, 12,000 pregnant women were infected with ZIKA and there were no reported microcephaly evidence in their babies as of May 2016 (Rasmussen et al., 2016). However, in April 2016, the CDC declared ZIKA the cause of microcephaly in Brazil (Rasmussen et al., 2016), yet outside of Brazil a similar number of cases have not been reported. In June 15, 2016, the WHO reported seven ZIKA associated microcephaly cases in Colombia and 1500 confirmed cases in Brazil (WHO, 2016). The results of a study in French Polynesia provided the evidence that 1 in 100 pregnancies exposed in the first and second trimester, resulted in microcephaly (Cauchemez et al., 2016). However, for ZIKA, asymptomatic infection is common, and only 20% of infected humans show symptoms like acute fever, maculopapular rashes, conjunctivitis, and arthralgia. Because of low mortality and mild symptoms, hospitalization ratio is relatively lesser as compared to Ebola like infections (Wojda et al., 2015).

The disease can be diagnosed by patient’s clinical signs and symptoms, resembling to other mosquito-borne viral diseases. The complete travel history of the patient to the infected areas can lead the physicians to correct diagnosis. Although the mosquito has been designated as the primary vector for ZIKA transmission, other modes of transmission like blood transfusion and sexual intercourse are also under consideration (Musso et al., 2015).

While the clinical differential diagnosis is not specific, diagnosis can be authenticated by performing a reverse transcriptase-polymerase chain reaction (RT-PCR) according to the CDC-issued guidance. The ZIKA RNA is detectable in serum after 7th days post-symptom onset. ZIKA RNA has been detected in serum in non-pregnant patients and pregnant patients after 62 days post-symptom onset and 53 days of after last known exposure in an asymptomatic pregnant women (Driggers et al., 2016; Meaney-Delman et al., 2016). ZIKA has been detected from number of body fluids, including urine, blood, saliva, and amniotic fluid (Musso et al., 2014; Barzon et al., 2016; Hills et al., 2016). However, one of the reports suggest that urine samples are more useful for diagnosis of ZIKA infections (Gourinat et al., 2015). The virus specific immunoglobulin M (IgM) as well as neutralizing antibodies can also be detected after 1 week of infection, but these detected antibodies are not specific (Tappe et al., 2014). In addition, other rapid tests such as reverse transcription loop-mediated isothermal amplification (RT-LAMP) (Lee et al., 2011) and RNA-biosensors (Baeumner et al., 2002) could be used as a first step for virus detection. Therefore, there is a dire need to develop a bedside test for ZIKA, to enable the clinicians for effective diagnosis of the patients infected with the virus.

## Public Health Response to Epidemic

Currently, there is no specific vaccine for the preventive treatment of ZIKA infection. However, different groups of scientists are working world over to develop the ZIKA vaccine, and hopefully will be available in couple of years (Daily Mail, 2016). For the effective disease control and prevention, the surveillance system can be drawn by reviewing the models that have already been used against dengue and chikungunya fever, but it should not be limited to only these two viral diseases (Tami et al., 2016).

The public health approaches that will be implemented for prevention of ZIKA should be capable of acknowledging the urbanization of diseases spreading through animal associated with population explosion, international trade and easy modes of intercontinental transportations. There are five clear strategies delineated by the Weaver (2013) where the interventions by public health authorities can be made. The first strategy is to intercept the enzootic life cycle. In this strategy, it is advised to stop the vector growth in its native environment; however, this strategy would not be feasible for ZIKA as it is difficult to control the vector. Another limitation to the strategy is that there is no available vaccine for ZIKA that could be inoculated in primates. The second strategy is to reduce the exposure of vulnerable subjects to the vector, in case of humans, applying bed nets and mosquito repellents that can decrease the exposure. The third technique that can reduce the disease burden is to limit the vector/source to the urban population. This could be done through control via modulating the vectorial capacity of the *Aedes* mosquito. Limiting the travel to infected areas also minimizes the risk of ZIKA. Fourth strategy could be the most helpful as well as it is an active strategy where the vector reservoirs are eliminated. In the case of ZIKA, proper drainage can reduce the stagnant water reservoirs to inhibit uncontrolled reproduction of mosquitoes. Adequate garbage management could also be used to hinder the vector proliferation. The fifth intervention that can be helpful is to avoid the recurrence of the disease where humans can act as the source of the virus for infection in non-human primates like monkeys. Avoiding mosquito bites to infected humans could be the aiding strategy for the prevention of spill over (Weaver, 2013).

The intervention of public health authorities is important, as the current epidemic is not confined to single geographic location. Despite vector-based transmission, blood transfusion could also be a cause of the spread among the patients having co-morbidities. In the recent past, 3% of the blood donors in French Polynesia were screened positive for ZIKA using PCR (Aubry et al., 2015).

It has also been reported that the ZIKA was present in the semen (Musso et al., 2015), therefore leading to a possibility of transmission through the sexual intercourse. If it is a sexually transmitted disease, the approaches used to avoid sexual transmitted diseases can also be employed to avoid the transmission. The contact tracing used in other sexual transmitted disease can be used to identify the other infected patients (LaMotte, 2016).

## Prevention Strategies

Till date, there is no curative medicine for the ZIKA disease, so to contain the epidemic prophylactic measures are bifurcated into following approaches: control vector density and personal protection (Daily Mail, 2016).

### Control Vector Density

The key measure to interrupt the transmission of viruses is to control the vector density. One of such control measures is Integrated Vector Management (IVM) recommended by WHO. Using the IVM model, overall sustainability, efficacy and cost-effectiveness of the strategy can be improved. The Integrated Management Strategy for the Prevention and Control of Dengue (IMS – Dengue) could be a basic model for ZIKA. Therefore, participation and collaboration of different organizations would play a great role in exploring IVM model. It has been reported that the *A. aegypti* is found in wide range of larval habitats both natural and manmade. There is a critical need of consistent implementation of the three-pronged IVM Model (Gubler and Clark, 1996). Some of the important control measures should include the followings:

• Monitoring of household and common areas to eliminate vector breeding sites, such as water reservoirs and waste drainage pipes.
• Regular cleaning of garbage collection sites.
• Development of physical, biological, and/or chemical methods to control mosquito breeding.
• Use of appropriate insecticides as per WHO recommendations.
• Fumigation of cargoes at ports and borders to prevent transport of larva by various means of transportation.

In addition to the above control measure, one of the key control measures is to avoid contact with vector. Therefore, personal prevention is very important to avoid sickness. Patients suffering with ZIKA infection and their family members must be well-educated about the risk of transmission. Use of mosquito nets, wire-mesh doors and windows, skin protection, and use of repellents are some of the key prevention measures that need to be taken under consideration.

#### Environmental Management

There are different environmental intervention and managements to control the growth of the *Aedes* species. In this subclass, authorities should be enabled to implement reliable water supply management, proper cleaning and maintenance of water storage systems, adequate solid waste management systems, and desirable alterations in human behaviors and residence systems. This includes proper cleaning of streets, maintenance and modifications of buildings/structures as well as housing units such as use of mosquito screens/nets on windows and mosquito proofing of water storages (Sikka et al., 2016).

#### Introduction of Bacteria into the Mosquito Population

A bacteria known as *Wolbachia* is present in approximately 60% of insects, commonly known as world’s most common reproductive parasite in the world (EDP, 2016). *Wolbachia* reduces the mosquito-to-human transmission events, ultimately reducing the transmission of virus to the humans from mosquito; the introduction of *Wolbachia* to offspring through the female mosquito’s egg will facilitate the beneficial epidemiological effect of securing humans from the bites. When the males with *Wolbachia* will mate with normal female mosquitos, females fail to hatch eggs while on other hand, the infected *Wolbachia* females will hatch eggs and produce offspring that will carry the *Wolbachia* effect (Nguyen et al., 2015). At the start, the technique will have a very limited effect as there will be a very few *Wolbachia* infected mosquitos present, but with the time, number of *Wolbachia* containing mosquitos increase, the significant effect can be observed. This approach has shown significant results to control dengue; it may also contribute significantly to control the ZIKA epidemic by limiting the ZIKA transmission since both ZIKA and dengue share similar clinical manifestation (Lambrechts et al., 2015).

#### Genetic Tailoring of the Mosquito

Another technique under consideration is to genetically modify the mosquitoes and giving rise to the population of mosquitoes whose offspring are not able to survive. Reducing the population of mosquitoes, ultimately will reduce the mosquito’s bites to humans as well as the vulnerable primates. Especially, using *A. aegypti* OX513A that has previously used to control dengue spread and hopefully it will help in controlling ZIKA spread as well (Phuc et al., 2007). *A. aegypti* OX513A is a genetically engineered strain, which is effective due to presence of release of insects carrying a dominant lethal (RIDL) genetic system (Alphey and Alphey, 2014). Similarly, offspring of OX513A, so the wild females will also die; this will keep the threshold of population needed for the disease spread. There are many benefits of the genetically modified vectors, but the most important fact is the dissemination of knowledge regarding capabilities of genetically modified vector to the concerned governmental officials, public health officials, and scientists (Alphey and Alphey, 2014).

Scientists and organizations are working on the genetic modification of the vectors and aware of the fact that transgenic technologies are associated with several environmental and safety concerns that still need to be addressed. There are several ecological side effects like unintended spread to non-target species; and horizontal transfer of the transgenes that should be properly addressed to avoid any undesired outcome (Gabrieli et al., 2014).

### Personal Prevention Measure

In the area of known ZIKA infection outbreak, the patients should avoid further contact with the vector to limit the spread of virus to other healthy people in the community. The community members should go through a proper awareness and education regarding prevention. The community members should be encouraged to act on the steps mentioned in **Table 2**. Insect repellents like *N, N*-diethyl-3-methylbenzamide, 3-(*N*-butyl-*N*-acetyl) amino propionic acid ethyl-ester or icaridin can be used. There are no specific recommendations and restrictions regarding use of these mosquito repellents, unless there is any specific warning given on the label of the product (Gibbons, 2002).

**Table 2:** Prevention recommendation (copied from Sikka et al., 2016).

Strategy	Action
**Control vector design**	• Diligent management and control of environmental factors.
	• Eliminate or reduce vector breeding sites in common areas.
	• Conduct mass sanitation campaigns to educate the public.
	• Ensure Mosquitoes are removed within the predetermined radius of critical places like schools, hospitals, transport terminals, using risk stratification paradigms.
	• In areas with viral activity, use mosquito adulticidal sprays to interrupt ZIKA transmission.
	• Ensure proper monitoring and follow-up during integrated actions for vector control.

**Preventative measure**	Individual protection
	• Encourage Individuals to use Bed-nets.
	• Appropriate clothing to cover exposed skin.
	• Use repellents.
	Household/residential protection
	• Encourage Installation and use of wire-mesh screens on doors and windows.
	• Once per week emptying, cleaning, turning over, and disposal of containers that can hold water inside or outside the houses to reduce any mosquito breeding sites.

In the first week of ZIKA infection, following preventive measures should be followed:

• *Aedes* mosquito bites should be avoided.
• The patients are advised to stay under the bed-nets.
• Another community that is under great risk of getting infected from the patients is the health workers. It is essential to protect the health workers so that other hospitalized patients might not get infected from the workers. In addition, care should be taken during blood donations and organ donations.
• Avoid sexual intercourse when traveling to infected area or when one of the partner is infected with ZIKA.
• Pregnant women are advised not to visit the setting where patients are resided. Similarly, pregnant women are advised not to visit where the epidemic is present.

### Vaccine Preparation

Multiple firms are looking to prepare ZIKA vaccine so this virus can be cured and does not ail our next generations. In Japan, Takeda Pharmaceutical, Co. Ltd has created a team to investigate the propensity for creating a vaccine (Reuters, 2016). In India, Bharat Biotech is looking in creating vaccine for the virus. It is reported that two possible vaccines are under process in India. In USA, Johnson and Johnson, Pfizer Inc. and Merck & Co. Inc. are evaluating the tendency of previous vaccines to combat ZIKA. Sanofi SA from France also launched a program to create ZIKA vaccine. All in all, pharmaceutical firms around the world are trying to create a vaccine for this virus, suggesting that a ZIKA vaccine is at ground zero. Till then preventive measures is the best way to avoid this virus (Reuters, 2016).

A group of researchers performed a drug repurposing screen of around 6000 compounds and identified some novel compounds that either suppress or inhibit ZIKA infection-induced caspase-3 activity in different neural cells. In addition, 10 different inhibitors of cyclin-dependent kinases inhibited ZIKA replication (Xu et al., 2016). One of these compounds are already existing drug, Niclosaminde and another potentially active against ZIKA is PHA-690509 (Xu et al., 2016). The results are still preminary and after successfull animal trails might be available to humans.

In another study, ZIKA-117, an antibody derived from the blood of ZIKA infected people potentially protect developing fetuses from the ravages of the ZIKA virus in mice. The antibody treatment inhibit the virus in the mother and protect the fetus (Sapparapu et al., 2016). However, development of ZIKA vaccine would be promising for long-lasting immunity against the virus than short-term antibody treatment.

## Brief History and Travelers Recommendations

**FIGURE 3:**
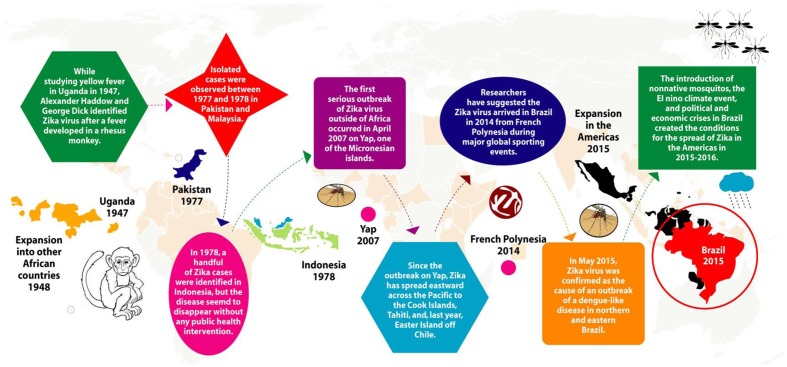
**Journey of ZIKA from Uganda to the Americas**.

In 1947, Alexander Haddon and George Dick first identified ZIKA in monkey while studying yellow fever in the ZIKA forest of Uganda. Subsequently, the same virus was isolated from the *Aedes* species of mosquitoes in the same forest of Uganda. In 1950, antibodies against ZIKA were detected in humans, and in 1968, ZIKA was isolated from humans in Nigeria. With time, the ZIKA spread to other parts of Africa as well as Asia. However, until 2007, no cases of ZIKA infection was found outside Africa and Asia, except an outbreak in Yap Island and French Polynesia in 2013. In 2015, the first case of ZIKA infection was detected in Brazil and has speak to more than 50 countries in the Americas (**Figure 3**).

Since there is a great risk of ZIKA infection due to travelers traveling from ZIKA infected regions to other non-infected regions, necessary precautions need to be taken. At present, there is no recommendation from the WHO as to travel bans to the affected countries. However, it is the government responsibility to educate the public while traveling to the areas known for epidemic spread (**Table 3**). Travelers should be advised to carry necessary measures if traveling to ZIKA stricken regions. In addition, appreciate information about the symptoms of ZIKA infection and prevention should be handy at airports, bus terminals, railways stations, and so on. This information could be also printed on travel documents such as air tickets or webpages. It is very important that upon returning from ZIKA prone areas, travelers should contact their healthcare providers before returning home.

**Table 3:** Recommendations to travelers (copied from Sikka et al., 2016).

Traveler status	Recommendations
**Prior to departure**	• Travelers are advised to protect themselves from mosquito bites during stay.
	• Use Mosquito repellents, wear appropriate clothing to minimize skin exposure.
	• Use insecticides and bed-nets.
	• Educate travelers about signs and symptoms of ZIKA/dengue/chikungunya virus in order to identification and to reduce the time to required medical attention.

**During visit**	• Avoidance of mosquito-infested areas.
	• Avoidance of mosquito bites.
	• Proactive and proper use of bed-nets and/or insecticide.
	• Seek professional care in case there are symptoms of ZIKA/dengue/Chikungunya

**Upon return**	• Travelers should contact appropriate health care provider in case ZIKA infection is suspected. Due to some symptomatic overlap, this is also applies to dengue and chikungunya viruses.

## Conclusion

ZIKA with no doubt took the world as a storm because of *Aedes* mosquitoes in South America. The virus entered and propagated in the country with conditions favorable for ZIKA like high population density, lack of immunity in targets and viral mutations. Global warming is a contributor in widespread of ZIKA as well (Ai et al., 2016). On the face of earth, no country is safe form ZIKA until preparation of vaccine. Countries having *Aedes* mosquito is on high alert, every day a new case emerges from other part of the world. ZIKA is progressing very fast and next few months are very crucial for stopping the attacks of *Aedes* mosquitos. High population directly affect the cases of ZIKA. So, if the virus outbreaks in those countries like India, China and USA, it would be impossible to control this virus. The current most workable suggestion to pregnant mothers is to avoid traveling to ZIKA affected areas. This is the best chance we have got till now, more research is necessary on this subject to find the cure for the disease. Cases of ZIKA has to be analyzed in detail to check the relationship of ZIKA and genetic mutation of *Aedes* to help understand why ZIKA is carried out in this type of mosquito (CDC, 2016b).

When ZIKA was identified, it caught us by surprise and no time could be leveraged to do anything in stopping this virus but there are major implications for researchers and doctors in studying ZIKA. It is still unidentified that how many more viruses of this type are present in our atmosphere (Lambrechts et al., 2015). Due to globalization, ZIKA can land anywhere through any channel. Global climate change and urban crowding also give way for ZIKA to grow. Maybe it is time when we need to rethink our public health infrastructure and disease-control strategies.

## Author Contributions

IR wrote the initial draft of the paper, designed figures and SK updated and proofread the paper. VB and JL did the critical analysis of the data and Y-HP designed, analyzed and approved the paper.

## Conflict of Interest Statement

The authors declare that the research was conducted in the absence of any commercial or financial relationships that could be construed as a potential conflict of interest.
